# Potential Drug-Drug Interactions in Patients With Urinary Tract Infections: A Contributing Factor in Patient and Medication Safety

**DOI:** 10.3389/fphar.2019.01032

**Published:** 2019-09-17

**Authors:** Sidra Noor, Mohammad Ismail, Fahadullah Khan

**Affiliations:** Department of Pharmacy, University of Peshawar, Peshawar, Pakistan

**Keywords:** patient safety, potential drug-drug interactions, urinary tract infections, clinical relevance, polypharmacy, adverse drug effects

## Abstract

**Introduction:** Hospitalized patients with urinary tract infections (UTIs) often present with comorbid illnesses and are subsequently prescribed multiple medications, which increases the likelihood of drug-drug interactions. Therefore, this study aimed to explore the prevalence, levels, risk factors, and clinical relevance of potential drug-drug interactions (pDDIs) in hospitalized patients with UTIs. Secondly, we aimed to develop management guidelines and identify monitoring parameters for the most frequent interactions.

**Methods:** A retrospective cross-sectional study was conducted in internal medicine wards of two tertiary care hospitals in Peshawar, Khyber Pakhtunkhwa, Pakistan. The clinical profiles of 422 patients with UTIs were reviewed for pDDIs using the Micromedex Drug-Reax^®^. Logistic regression was applied to assess the association of pDDIs with various risk factors. The clinical relevance of frequent pDDIs was identified by assessing the potential adverse outcomes of pDDIs including patients’ signs, symptoms, and abnormal laboratory findings.

**Results:** Of 422 patients, at least one pDDI was identified in 62.3% patients, while 40% patients had at least one major pDDI. A total of 1,086 pDDIs were identified, of which 53.4% and 39.3% were of moderate and major severity, respectively. Patients with most frequent pDDIs were presented with hypoglycemia, hepatotoxicity, nephrotoxicity, hypertension, and decreased therapeutic response. These adverse events were more prevalent in patients taking higher doses of interacting drugs. Multivariate regression analysis revealed significant association of pDDIs with six or more medicines (*p* < 0.001), diabetes mellitus (*p* < 0.001), ischemic heart disease (*p* = 0.02), and congestive cardiac failure (*p* = 0.04).

**Conclusions:** Patients with UTIs present with a considerable number of clinically important pDDIs. Polypharmacy, diabetes mellitus, ischemic heart disease, and congestive cardiac failure increase the risk of pDDIs. Knowledge about the most frequent pDDIs will enable healthcare professionals to implement optimized monitoring and management strategies regarding associated adverse consequences in order to ensure patient safety. Most of the interactions can be managed by considering alternative therapy and dose reduction.

## Introduction

Urinary tract infections (UTIs) are among the major health problems that affect millions of people (Foxman, 2010; Blondal et al., 2016). Each year, in the United States UTIs account for nearly seven million clinic visits, one million emergency visits, and 100,000 hospital admissions (Schappert, 1999).

Patients with UTIs are hospitalized due to the severe nature of the disease, comorbid illnesses, and associated complications (Briongos-Figuero et al., 2012). Such patients are usually prescribed with antipyretics and antibiotics including cephalosporins, aminoglycosides, and quinolones (Dhodi et al., 2014; Panayappan et al., 2017). Apart from the use of these drugs, a large number of other drugs are also prescribed in order to treat the associated symptoms and comorbid illnesses (Dhodi et al., 2014). The simultaneous use of such large number of drugs increases the risk of drug-drug interactions (DDIs) by altering the pharmacokinetic parameters or the pharmacodynamic profile of drugs (Zwart-van-Rijkom et al., 2009; Juurlink et al., 2013). DDIs may lead to a number of undesirable consequences such as decreased or abolished clinical effectiveness, adverse effects, hospitalization, and prolongation of hospital stay (Ray et al., 2004; Juurlink et al., 2013; Khan et al., 2017). DDIs account for 20–30% of adverse effects, of which 70% require clinical intervention and 1–2% are life-threatening (Kohler et al., 2000). Hence, proper consideration of DDIs and their timely management is essential for the safe and effective use of medicines among patients with UTIs.

Studies have addressed the issue of potential DDIs (pDDIs) in hospitalized patients (Zwart-van-Rijkom et al., 2009) as well as in specific clinical specialties such as oncology (Van-Leeuwen et al., 2013), cardiology (Murtaza et al., 2016; Khan et al., 2017), psychiatry (Hahn et al., 2013), and internal medicine (Vonbach et al., 2007; Ismail et al., 2013). Moreover, some studies have investigated pDDIs among patients with specific infectious diseases such as hepatitis C and acquired immune deficiency syndrome (Patel et al., 2011; Kondili et al., 2017; Langness et al., 2017) as well as pDDIs in patients with chronic diseases such as liver cirrhosis (Franz et al., 2012), heart failure (Straubhaar et al., 2006), hypertension (Subramanian et al., 2018), stroke (Caratozzolo et al., 2016), and hematopoietic stem cell transplantation (Trevisan et al., 2015). However, this issue remains unaddressed among patients with UTIs, despite being a frequent cause of hospitalization (Ismail et al., 2013). Additionally, the irrational use of drugs and scarcity of literature is common in developing countries. Therefore, studies are needed regarding various aspects of pDDIs and their clinical relevance among hospitalized patients with UTIs. Consequently, such studies will improve patients’ safety, achieve positive clinical outcomes, and help healthcare professionals to manage pDDIs and reduce their associated problems.

This study aimed to explore the prevalence, levels, risk factors, and clinical relevance of pDDIs in hospitalized patients with UTIs. Secondly, the study aimed to develop management guidelines and identify monitoring parameters for the most frequent interactions.

## Methods

### Study Settings and Design

This was a retrospective cross-sectional study, conducted in the internal medicine wards of two tertiary care hospitals in Peshawar, Khyber Pakhtunkhwa, Pakistan: Hayatabad Medical Complex and Khyber Teaching Hospital. Clinical pharmacy services and computerized drug interaction screening programs do not exist in both hospitals. Patients’ profiles are maintained in handwritten format using predefined charts.

### Inclusion and Exclusion Criteria

The inclusion criteria were the following:

- Patients diagnosed with UTIs and admitted to internal medicine wards.
- Age ≥18 years.
- Both male and female patients.

Profiles were excluded if they were incomplete with respect to relevant data required for this study.

### Data Source

Based on the above criteria, we included 422 patients’ profiles. Administrative permission from both hospitals was obtained in order to access patients’ clinical records. Data regarding hospital admissions, patients’ demographics, diagnoses, comorbidities/complications, medication therapy, signs/symptoms, and laboratory tests were collected.

### Screening of Medication Profiles for pDDIs

All medications prescribed during hospitalization (from the time of admission till discharge) were evaluated for pDDIs using the Micromedex Drug-Reax^®^ (Micromedex Drug-Reax, 2017). This software classifies drug interactions on the basis of severity and documentation levels as follows (Micromedex Drug-Reax, 2017):

Severity Levels:

- Contraindicated: Concurrent use of the interacting pair is contraindicated.
- Major: The interacting pair may result in permanent damage/death; medical intervention is needed to prevent or minimize the adverse outcome.
- Moderate: The combination may worsen the patient’s condition and/or require an alteration in therapy.
- Minor: There are limited clinical effects of the interaction. These may include an increase in the severity or frequency of adverse effects, and major alteration of therapy is not required.

Documentation Levels (Scientific Evidence):

- Excellent: Controlled studies have demonstrated the existence of interaction.
- Good: Well-controlled studies are lacking, but documentation strongly suggests the existence of interaction.
- Fair: Existing documentation is less, but physicians suspect the presence of interaction on the basis of pharmacological considerations, or evidences are good for interactions involving pharmacologically similar drug.

The overall prevalence of pDDIs and prevalence based on the severity levels (contraindicated, major, moderate, and minor) have been reported. Levels (severity and documentation) of pDDIs were also identified.

### Clinical Relevance

The clinical relevance of the 10 most frequent pDDIs was identified by assessing the potential adverse outcomes of pDDIs including patients’ signs, symptoms, and abnormal laboratory findings. The clinical features have been stratified based on dosage variations of the interacting drugs. The following cutoff points were used for defining higher daily doses: aspirin: ≥150 mg; nitroglycerin: ≥5.2 mg; ramipril: ≥10 mg; bisoprolol: ≥10 mg; furosemide: ≥60 mg; isoniazid: ≥150 mg; rifampin: ≥300 mg; and pyrazinamide: ≥500 mg. In this study, adverse drug events were defined as follows: hypoglycemia: random blood sugar <80 mg/dl or fasting blood sugar <70 mg/dl; hypertension: systolic blood pressure (BP) >130 mmHg or diastolic BP >80 mmHg; hypotension: systolic BP <90 mmHg or diastolic BP <60 mmHg; tachycardia: heart rate >100 beats/ min; bradycardia: heart rate <60 beats/min; hypokalemia: serum potassium <3.5 mmol/L; hyponatremia: serum sodium <135 mmol/L; hypernatremia: serum sodium >145 mmol/L; hypochloremia: serum chloride <95 mmol/L; hyperchloremia: serum chloride >105 mmol/L; leukocytosis: total leukocyte count >11,000/μl; neutrophilia: neutrophil count >76%; decreased platelets counts: <150,000/μl; increased alkaline phosphatase: >126 U/L; increased serum bilirubin: >1 mg/dl; increased alanine aminotransferase: >59 U/L (male), >36 U/L (female). Monitoring parameters and management guidelines were described for the most frequent interactions. A list of the clinically important pDDIs (based on severity levels) was developed along with their potential adverse outcomes.

### Statistical Analysis

Descriptive statistics were used for presenting data in the form of frequencies and percentages with median and interquartile range (IQR), where appropriate. Logistic regression analysis was applied in order to identify association for one or more pDDIs with patients’ characteristics. Moreover, association for major pDDIs with patients’ characteristics was also identified. Dependent variables in the model were exposure to all types- or major pDDIs, while patients’ characteristics were independent variables. For each independent variable, odds ratios (OR) and 95% confidence intervals (CI) were determined. Initially, the univariate logistic regression analysis was carried out. Then, for variables with *p* values of <0.15, multivariate analyses were performed. We considered, *p* value of <0.05 as statistically significant. SPSS-v23 was used for statistical analyses of the data.

## Results

### Patients’ General Characteristics

Patient demographics and comorbidities are shown in **Table 1**. Of a total of 422 patients, 284 (67.3%) were female. The median age was 55 years (IQR = 41–65), median prescribed drugs were 9 (7–12), and median hospital stay was 5 days (3–7). Majority of patients were aged 18–60 years (72%). Most of the patients were prescribed with 6–10 medicines (47.6%). Diabetes mellitus (DM) (*n* = 212; 50.2%), hypertension (191; 45.3%), hepatitis (45; 10.7%), chronic kidney disease (CKD) (42; 10%), and ischemic heart disease (IHD) (41; 9.7%) were the most frequent comorbidities.

**Table 1:** General characteristics of study subjects (*n* = 422).

Characteristic	Patients: N (%)
**Gender**	
Male	138 (32.7)
Female	284 (67.3)
**Age (years)**	
18–60	304 (72)
>60	118 (28)
Median (IQR)	55 (41–65)
**Drugs prescribed per patient**	
≤5	63 (14.9)
6–10	201 (47.6)
>10	158 (37.4)
Median (IQR)	9 (7–12)
**Hospital stay (days)**	
≤3	105 (24.9)
4–6	196 (46.4)
>6	121 (28.7)
Median (IQR)	5 (3–7)
**Comorbidities**	
Diabetes mellitus	212 (50.2)
Hypertension	191 (45.3)
Hepatitis	45 (10.7)
Chronic kidney disease	42 (10)
Ischemic heart disease	41 (9.7)
Stroke	30 (7.1)
Respiratory tract infection	23 (5.5)
Congestive cardiac failure	21 (5)
Decompensated chronic liver disease	20 (4.7)
Anemia	16 (3.8)
Acute gastroenteritis	15 (3.6)
Pneumonia	14 (3.3)
Rheumatic arthritis	12 (2.8)
Malaria	11 (2.6)
Tuberculosis	11 (2.6)
Cholelithiasis	9 (2.1)
Chronic obstructive pulmonary disease	7 (1.7)
Hepatic encephalopathy	7 (1.7)
Meningitis	7 (1.7)
Myeloma	6 (1.4)
Miscellaneous	145 (34.4)

IQR, interquartile range.

### Prevalence of pDDIs

**Figure 1:**
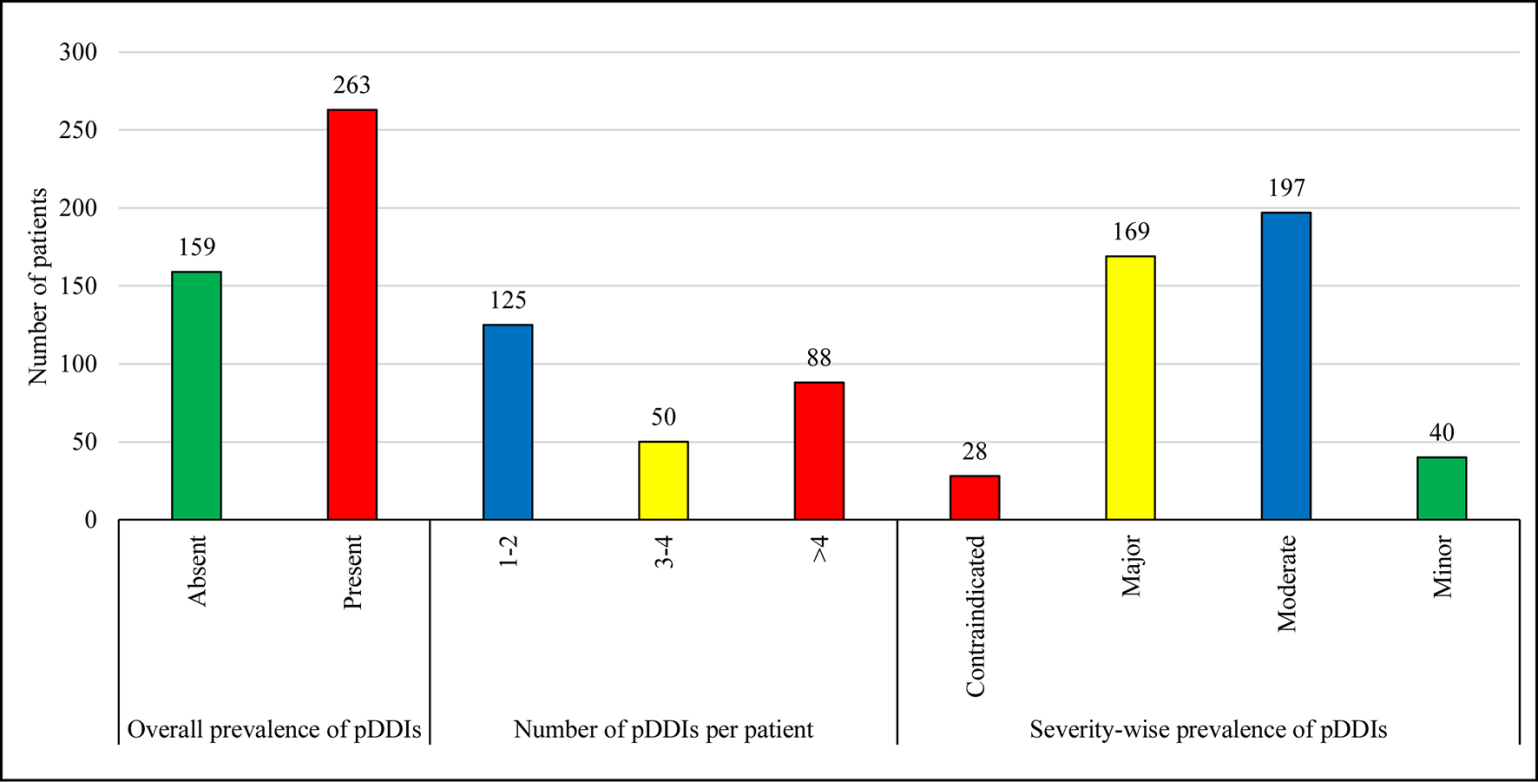
Overall-prevalence is the occurrence of at least one pDDI irrespective of severity type. The total number of UTI patients was 422. Therefore, the overall prevalence of pDDIs is 62.3% (263 out of 422). Data are presented in the form of frequencies. The prevalence of pDDIs has also been reported on the basis of severity levels. pDDIs, potential drug-drug interactions.

**Figure 1** illustrates the prevalence of pDDIs. Of a total of 422 patients, 62.3% were exposed to at least one pDDI. Based on severity-wise prevalence, 46.7% and 40% patients were presented with pDDIs of moderate and major severity, respectively, while the prevalence of contraindicated and minor severity were observed less frequently.

### Levels of Potential Drug-Drug Interactions

**Figure 2:**
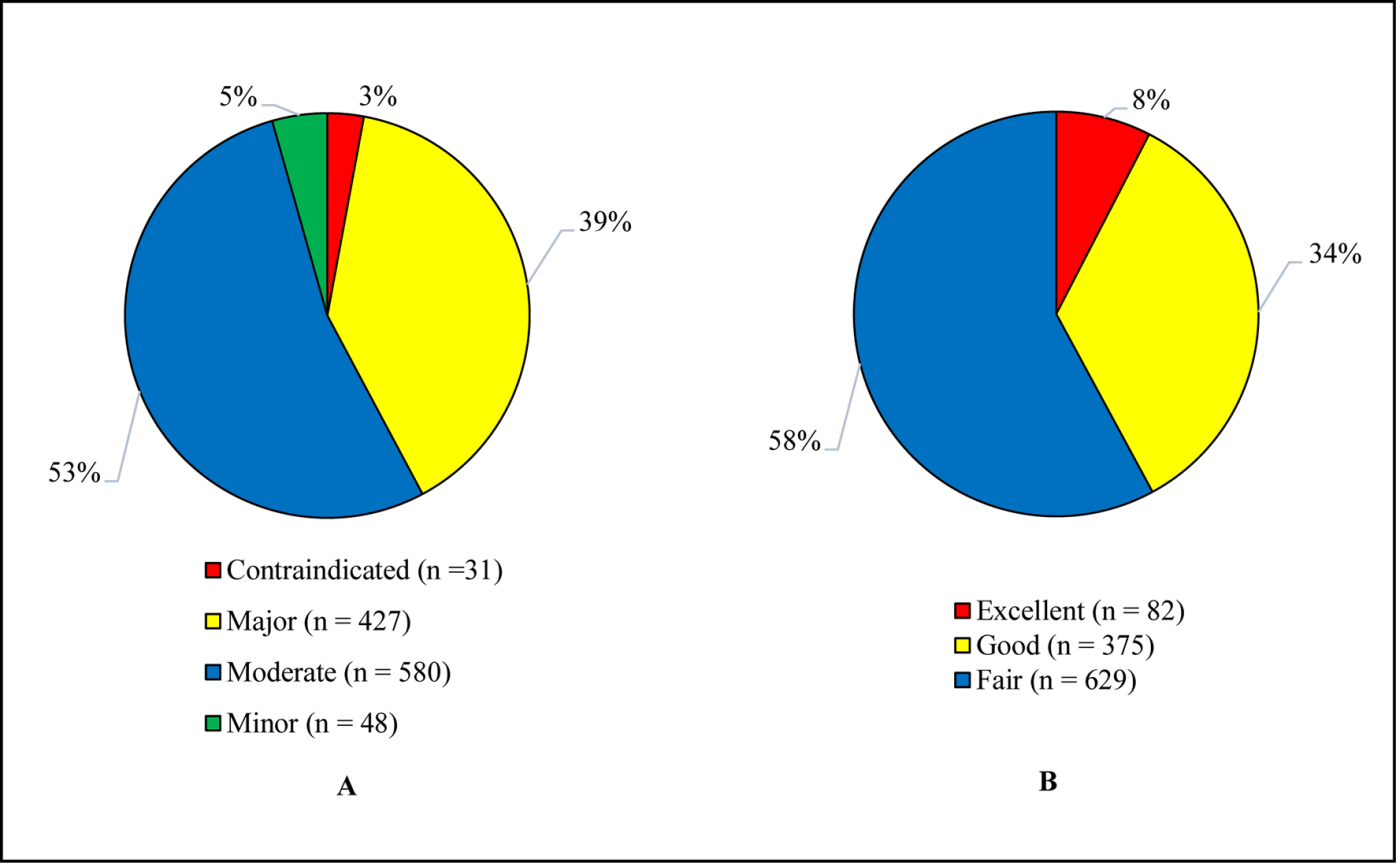
**(A)** Severity levels of pDDIs. **(B)** Documentation levels of pDDIs. The total identified pDDIs were categorized based on the severity and documentation levels. pDDIs, potential drug-drug interactions.

**Figure 2** illustrates the levels of pDDIs. The recorded pDDIs were categorized on the basis of severity and documentation levels. A total of 1,086 interactions were identified, of which 53.4% were of moderate and 39.3% major severity, whereas 57.9% and 34.5% were about fair and good scientific evidence, respectively.

### Risk Factors of Potential Drug-Drug Interactions

Multivariate logistic regression analysis showed significant association of all types of pDDIs with six to eight prescribed medicines (OR = 7; *p* < 0.001), eight or more prescribed medicines (OR = 32; *p* < 0.001), DM (OR = 2.8; *p* < 0.001), and IHD (OR = 4.3; *p* = 0.02). Similarly, there was a significant association of major pDDIs with six to eight prescribed medicines (OR = 7.7; *p* = 0.009), eight or more prescribed medicines (OR = 40; *p* < 0.001), IHD (OR = 3; *p* = 0.01), and congestive cardiac failure (CCF) (OR = 3.3; *p* = 0.04) as presented in **Table 2**.

**Table 2:** Logistic regression analysis based on exposure to all types and major interactions.

Variables	All types of interactions				Major interactions			
	Univariate analysis		Multivariate analysis		Univariate analysis		Multivariate analysis	
	OR (95% CI)	*p* value	OR (95% CI)	*p* value	OR (95% CI)	*p* value	OR (95% CI)	*p* value
**Gender**								
Male	Reference		Reference		Reference		–	
Female	1.6 (1-2.4)	0.03	0.9 (0.6-1.7)	1	1.3 (0.9-2)	0.2	–	–
**Age (Years)**								
18-60	Reference		–		Reference		–	
>60	1.2 (0.8-1.9)	0.4	–	–	1.2 (0.8-1.8)	0.4	–	–
**Drugs prescribed**								
≤5	Reference		Reference		Reference		Reference	
6-8	5.7 (2.5-12.9)	<0.001	7 (2.8-17.2)	<0.001	6.4 (1.4-28.4)	0.01	7.7 (1.7-35.2)	0.009
>8	34 (15.1-76.8)	<0.001	32 (13-81.5)	<0.001	46.2 (11-193)	<0.001	40 (9-174)	< 0.001
**Hospital stay (days)**								
≤3	Reference		Reference		Reference		Reference	
4-6	2.3 (1.4-3.7)	0.001	1.1 (0.6-2.1)	0.7	1.7 (1-2.9)	0.04	0.8 (0.4-1.5)	0.4
>6	2.6 (1.5-4.5)	0.001	0.9 (0.5-2)	0.9	3.3 (1.9-5.8)	<0.001	1.3 (0.7-2.7)	0.4
**Comorbidities**								
Diabetes mellitus	3.8 (2.5-5.8)	<0.001	2.8 (1.6-4.7)	<0.001	2.3 (1.6-3.5)	<0.001	1.4 (0.8-2.3)	0.2
Hypertension	2.5 (1.7-3.8)	<0.001	1 (0.6-1.9)	0.9	2.4 (1.6-3.5)	<0.001	1.3 (0.8-2.2)	0.3
Hepatitis	0.9 (0.5-1.9)	0.9	–	–	0.9 (0.5-1.7)	0.7	–	–
Chronic kidney disease	1.8 (0.9-3.7)	0.10	0.8 (0.3-1.8)	0.6	1.4 (0.7-2.7)	0.3	–	–
Ischemic heart disease	4.9 (1.9-12.7)	0.001	4.3 (1.3-15)	0.02	4.2 (2.1-8.4)	<0.001	3 (1.3-7)	0.01
Stroke	2.6 (1-6.4)	0.04	1.7 (0.6-5.3)	0.3	1.8 (0.8-3.8)	0.13	1.1 (0.4-2.7)	0.9
Respiratory tract infection	2.3 (0.8-6.2)	0.11	1.7 (0.5-5.5)	0.4	1.7 (0.7-3.9)	0.2	–	–
Congestive cardiac failure	3.8 (1.1-13.1)	0.03	1.4 (0.4-5.5)	0.6	6.9 (2.3-21)	0.001	3.3 (1-10.6)	0.04
Decompensated chronic liver disease	1.4 (0.5-3.8)	0.5	–	–	1.5 (0.6-3.8)	0.4	–	–
Anemia	0.5 (0.2-1.2)	0.13	0.4 (0.1-1.3)	0.1	0.7 (0.2-1.9)	0.5	–	–

CI, confidence interval; OR, odds ratio.

### Clinical Relevance of Potential Drug-Drug Interactions

The prescribed dosage of the interacting drugs is shown in **Table 3**. The drugs were given in a variety of doses and administration frequencies. However, most of the patients received low doses of the following interacting drugs: aspirin, ramipril, bisoprolol, furosemide, ceftriaxone, isoniazid, rifampin, and pyrazinamide. Higher doses of the interacting drugs were comparatively less frequent for the following drugs: insulin, metformin, nitroglycerin, ceftriaxone, isoniazid, rifampin, and pyrazinamide. See **Supplementary Table 1** for the most frequent interactions. Most frequently prescribed antimicrobial agents (AMAs) and drugs besides AMAs are listed in **Supplementary Table 2** and **Table 3**, respectively.

**Table 3:** Prescribed dose regimen of the interacting drugs.

Interacting pair	Dose categories^a^	Prescribed dose regimen	Number of patients
Aspirin + Insulin	Low + High	75mg OD + 24-80 units per day	29
	Low + Low	75mg OD + 8-20 units per day	7
	High + High	150mg OD + 24-50 units per day	3
	High + High	300mg OD + 30 units per day	3
		
Insulin + Metformin	High + High	21-80 units per day + ≥850mg OD/BD	13
	High + Low	21-50 units per day + 500mg OD	8
	High + High	21-72 units per day + 500mg BD	8
	Low + High	≤20 units per day + 500mg BD	2
	Low + Low	≤20 units per day + ≤500mg OD	1
	Low + High	≤20 units per day + ≥850mg OD	1
		
Insulin + Ramipril	High + Low	21-50 units per day + 5mg OD	10
	High + Low	21-50 units per day + 2.5mg OD	8
	High + High	21-60 units per day + 10mg OD	4
	Low + Low	≤20 units per day + 5mg OD	2
		
Aspirin + Nitroglycerin	Low + High	75mg OD + 2.6mg BD	13
	High + High	300mg OD + 2.6mg BD	5
	Low + Low	75mg OD + 0.5mg OD	1
	High + Low	150mg OD + 0.5mg OD	1
	High + High	150mg OD + 2.6mg BD	1
		
Aspirin + Ramipril	Low + Low	75mg OD + 5mg OD	7
	Low + Low	75mg OD + 2.5mg OD	6
	Low + High	75mg OD + 10mg OD	4
	High + Low	300mg OD + 2.5mg OD	2
	High + Low	300mg OD + 5mg OD	1
Aspirin + Bisoprolol	Low + Low	75mg OD + 5mg OD	7
	Low + Low	75mg OD + 2.5mg OD	5
	High + Low	300mg OD + 2.5mg OD	3
	High + Low	150mg OD + 2.5mg OD	2
	High + Low	150mg OD + 5mg OD	1
	Low + High	75mg OD + 10mg OD	1
	High + Low	300mg OD + 5mg OD	1
Aspirin + Furosemide	Low + Low	75mg OD + 40mg OD	8
	Low + Low	75mg OD + 20mg OD	4
	Low + High	75mg OD + 40mg BD	4
	Low + High	75mg OD + 60mg OD	1
	Low + High	75mg OD + 60mg BD	1
	Low + High	75mg OD + 80mg OD	1
	High + Low	300mg OD + 40mg OD	1
		
Calcium containing products + Ceftriaxone	Low + Low	200mg/L BD + 1gm BD ATD	6
	Low + Low	200mg/L BD + 2gm OD ATD	5
	Low + High	200mg/L BD + 3gm OD ATD	3
	Low + High	200mg/L BD + 2gm BD ATD	3
	High + High	1gm + 2gm BD ATD	1
	Low + High	169mg OD + 2gm BD ATD	1
		
Isoniazid + Rifampin	Low + Low	75mg OD + 150mg OD	15
	High + High	150mg OD + 300mg OD	4
		
Pyrazinamide + Rifampin	Low + Low	400mg OD + 150mg OD	15
	High + High	500mg OD + 300mg OD	4

ATD, alternate day; BD, twice a day; OD, once a day.

a The following cut off points were used for defining higher daily doses, aspirin: ≥150mg; nitroglycerin: ≥5.2mg; ramipril: ≥10mg; bisoprolol: ≥10mg; furosemide: ≥60mg; isoniazid: ≥150mg; rifampin: ≥300mg; and pyrazinamide: ≥500mg.

Relevant clinical findings in low- and high-dose groups for the 10 most frequent pDDIs are presented in **Table 4**. Patients with the interactions aspirin + insulin, insulin + metformin, and insulin + ramipril were presented with signs/symptoms and abnormal laboratory findings indicating hypoglycemia. Signs/symptoms of hypoglycemia were highly prevalent in the high-dose groups. Nitroglycerin toxicity and decreased antiplatelet response were more frequent in patients taking high doses of aspirin + nitroglycerin. Signs/symptoms suggesting poor response and electrolyte abnormalities were more frequent in the high-dose groups of the following interacting drugs: aspirin + ramipril, aspirin + furosemide, and calcium-containing products + ceftriaxone. Similarly, signs/symptoms of hypertension were highly prevalent among high-dose groups of aspirin + bisoprolol. In patients with interactions, isoniazid + rifampin and pyrazinamide + rifampin, signs/symptoms of hepatotoxicity such as anorexia, paleness, weight loss, abdominal pain, weakness, hepatomegaly, fatigue, and myalgia were observed. Similarly, abnormal laboratory findings such as increased alanine aminotransferase, alkaline phosphatase, and serum bilirubin were reported. These signs/symptoms were more prevalent among low-dose groups of isoniazid, rifampin, and pyrazinamide. Management guidelines and monitoring parameters have also been provided in **Table 4**. Adverse consequences for the most frequent pDDIs were reduced therapeutic efficacy, electrolyte abnormalities, hypoglycemia, hypertension, hepatotoxicity, bleeding, and hypotension.

**Table 4:** Clinical relevance of ten most frequent potential drug-drug interactions.

Interactions (N)	Dose categories (N)	Signs/symptoms and laboratory investigations	Patients: N (%^a^)	Management guidelines or monitoring parameters
Aspirin – Insulin (42)	Low + Low (7)	Drowsiness	2 (28.6)	Monitor the patient’s blood glucose, clinical signs of hypoglycemia and adjust the dose of insulin if necessary
		Tachycardia	2 (28.6)	
		Pale	2 (28.6)	
		Irregular heart rate	1 (14.3)	
		Dehydration	1 (14.3)	
		Headache	1 (14.3)	
		Confusion	1 (14.3)	
	Low + High (29)	Drowsiness	7 (24.1)	
		Tachycardia	5 (17.2)	
		Pale	5 (17.2)	
		Confusion	5 (17.2)	
		Fatigue	4 (13.8)	
		Irregular heart rate	4 (13.8)	
		Headache	3 (10.3)	
		Dehydration	3 (10.3)	
		Weakness	3 (10.3)	
		Palpitations	1 (3.4)	
		Loss of consciousness	1 (3.4)	
		Blurred vision	1 (3.4)	
		Shakiness	1 (3.4)	
	High + High (6)	Drowsiness	2 (33.3)	
		Tachycardia	2 (33.3)	
		Pale	2 (33.3)	
		Fatigue	2 (33.3)	
		Weakness	2 (33.3)	
		Headache	2 (33.3)	
		Palpitations	1 (16.6)	
		Depressive	1 (16.6)	
		Loss of consciousness	1 (16.6)	
Insulin – Metformin (33)	Low + Low (1)	Tachycardia	1 (100)	Monitor the patient’s blood glucose, clinical signs of hypoglycemia and adjust the dose of insulin if necessary
		Headache	1 (100)	
	High + Low (8)	Dehydration	2 (25)	
		Pale	2 (25)	
		Weakness	1 (12.5)	
		Loss of consciousness	1 (12.5)	
		Shakiness	1 (12.5)	
		Tachycardia	1 (12.5)	
	Low + High (3)	Drowsiness	1 (33.3)	
	High + High (21)	Tachycardia	5 (23.8)	
		Drowsiness	4 (19)	
		Weakness	4 (19)	
		Headache	3 (14.3)	
		Blurred vision	1 (4.8)	
		Confusion	1 (4.8)	
		Loss of consciousness	1 (4.8)	
		Fatigue	1 (4.8)	
		Decreased RBS	1 (4.8)	
Insulin – Ramipril (24)	Low + Low (2)	Pale	1 (50)	Monitor the patient’s blood glucose, clinical signs of hypoglycemia and adjust the dose of insulin if necessary
		Decreased FBS	1 (50)	
	High + High (4)	Pale	3 (75)	
		Drowsiness	1 (25)	
		Fatigue	1 (25)	
		Tachycardia	1 (25)	
		Irregular heart rate	1 (25)	
		Blurred vision	1 (25)	
		Dehydration	1 (25)	
		Palpitations	1 (25)	
	High + Low (18)	Tachycardia	7 (38.9)	
		Drowsiness	4 (22.2)	
		Dehydration	4 (22.2)	
		Headache	3 (16.6)	
		Weakness	2 (11.1)	
		Fatigue	2 (11.1)	
		Pale	2 (11.1)	
		Shakiness	1 (5.6)	
		Confusion	1 (5.6)	
		Irregular heart rate	1 (5.6)	
Aspirin – Nitroglycerin (21)	Low + High (13)	Hypotension	5 (38.5)	Analgesic doses of aspirin increase signs of nitroglycerin toxicity, patients should be monitored for hypotension, headache, and for signs of bleeding in patients with long term antiplatelet use of aspirin along with nitroglycerin
		Tachycardia	3 (23.1)	
		Pale	2 (15.4)	
		Drowsiness	2 (15.4)	
		Vertigo	1 (7.7)	
		Weakness	1 (7.7)	
		Headache	1 (7.7)	
		Fatigue	1 (7.7)	
		Decreased platelets	1 (7.7)	
	High + High (6)	Hypotension	4 (66.7)	
		Drowsiness	2 (33.3)	
		Tachycardia	2 (33.3)	
		Weakness	2 (33.3)	
		Pale	2 (33.3)	
		Fatigue	1 (16.7)	
		Depressed	1 (16.7)	
		Headache	1 (16.7)	
		Palpitations	1 (16.7)	
		Loss of consciousness	1 (16.7)	
		Dehydration	1 (16.7)	
		Decreased platelets	1 (16.7)	
	Low + Low (1)	Hypotension	1 (100)	
	High + Low (1)	Headache	1 (100)	
		Bradycardia	1 (100)	
		Hypertension	1 (100)	
Aspirin – Ramipril (20)	Low + Low (13)	Hypertension	9 (45)	Patients’ blood pressure, hemodynamic parameters, and renal function should be monitored. If an adverse effect is noted, the following options may be considered: (a) aspirin dosage less than 100 mg per day (b) an alternative non-aspirin antiplatelet agent (c) replacing ACE inhibitors with angiotensin receptor blockers
		Drowsiness	5 (25)	
		Tachycardia	5 (25)	
		Increased BUN	5 (25)	
		Increased serum creatinine	4 (20)	
		Hyponatremia	3 (15)	
		Fatigue	2 (10)	
		Headache	2 (10)	
		Irregular heart rate	1 (5)	
		Nausea	1 (5)	
		Chest pain	1 (5)	
		Hypernatremia	1 (5)	
		Hypokalemia	1 (5)	
		Confusion	1 (5)	
	High + Low (3)	Increased BUN	3 (100)	
		Increased serum creatinine	3 (100)	
		Irregular heart rate	1 (33.3)	
		Loss of consciousness	1 (33.3)	
		Headache	1 (33.3)	
		Chest pain	1 (33.3)	
		Tachycardia	1 (33.3)	
		Hyponatremia	1 (33.3)	
		Hypokalemia	1 (33.3)	
	Low + High (4)	Hypertension	4 (100)	
		Increased BUN	3 (75)	
		Hyponatremia	3 (75)	
		Increased serum creatinine	2 (50)	
		Nausea	2 (50)	
		Palpitations	1 (25)	
		Irregular heart rate	1 (25)	
		Tachycardia	1 (25)	
		Fatigue	1 (25)	
		Drowsiness	1 (25)	
		Blurred vision	1 (25)	
		Urinary retention	1 (25)	
Aspirin – Bisoprolol (20)	Low + Low (12)	Hypertension	8 (66.7)	Patients’ blood pressure and hemodynamic parameters should be monitored
		Nausea	3 (25)	
		Irregular heart rate	3 (25)	
		Drowsiness	3 (25)	
		Tachycardia	2 (16.7)	
		Palpitations	2 (16.7)	
		Headache	1 (8.3)	
		Confusion	1 (8.3)	
		Loss of consciousness	1 (8.3)	
	High + Low (7)	Hypertension	3 (42.9)	
		Loss of consciousness	3 (42.9)	
		Drowsiness	2 (28.6)	
		Nausea	2 (28.6)	
		Chest pain	2 (28.6)	
		Tachycardia	1 (14.3)	
		Headache	1 (14.3)	
		Fatigue	1 (14.3)	
		Palpitations	1 (14.3)	
		Irregular heart rate	1 (14.3)	
	Low + High (1)	Irregular heart rate	1 (100)	
		Headache	1 (100)	
		Tachycardia	1 (100)	
Aspirin – Furosemide (20)	Low + Low (12)	Increased BUN	9 (75)	Patients should be monitored for signs of renal toxicity and salicylate toxicity. Diuretic effectiveness should be assured including its effects on blood pressure. Avoid high dose of salicylates in those taking loop diuretics, an alternative analgesic should be given
		Hypertension	6 (12)	
		Increased serum creatinine	5 (41.7)	
		Hyponatremia	4 (33.3)	
		Pedal edema	3 (25)	
		Drowsiness	3 (25)	
		Fatigue	2 (16.7)	
		Chest pain	2 (16.7)	
		Confusion	2 (16.7)	
		Nausea	2 (16.7)	
		Hypokalemia	2 (16.7)	
		Hyperchloremia	1 (8.3)	
		Hypochloremia	1 (8.3)	
	Low + High (7)	Pedal edema	4 (57.1)	
		Increased BUN	3 (43)	
		Hyperchloremia	3 (43)	
		Hyponatremia	3 (43)	
		Hypertension	2 (29)	
		Hypokalemia	2 (29)	
		Drowsiness	2 (29)	
		Headache	2 (29)	
		Fatigue	2 (29)	
		Increased serum creatinine	2 (29)	
		Hypernatremia	1 (14.3)	
		Urinary retention	1 (14.3)	
		Ascites	1 (14.3)	
		Confusion	1 (14.3)	
	High + Low (1)	Chest pain	1 (100)	
		Increased BUN	1 (100)	
		Increased serum creatinine	1 (100)	
Calcium containing products – Ceftriaxone (19)	Low + Low (11)	Increased BUN	5 (45.4)	Do not mix or administer ceftriaxone concurrently with calcium-containing IV solutions in the same IV administration line, including continuous calcium-containing infusions such as parenteral nutrition via a Y-site. Monitor patient for signs of nephrotoxicity or decreased ceftriaxone effectiveness
		Leukocytosis	4 (36.3)	
		Increased serum creatinine	3 (27.3)	
		Neutrophilia	2 (18.2)	
		Fever	2 (18.2)	
	Low + High (7)	Fever	4 (57.1)	
		Increased serum creatinine	3 (43)	
		Leukocytosis	2 (29)	
		Neutrophilia	2 (29)	
		Increased BUN	2 (29)	
		Chest pain	1 (14.3)	
		Nephrolithiasis	1 (14.3)	
	High + High (1)	Increased BUN	1 (100)	
		Increased serum creatinine	1 (100)	
		Leukocytosis	1 (100)	
Isoniazid – Rifampin (19)	Low + Low (15)	Anorexia	4 (26.7)	Patients should be monitored for signs and symptoms of liver toxicity including fever, anorexia, vomiting and jaundice. Baseline and periodic LFTs monitoring is suggested
		Abdominal pain	4 (26.7)	
		Fever	3 (20)	
		Pale	3 (20)	
		Nausea	3 (20)	
		Weakness	3 (20)	
		Increased ALP	3 (20)	
		Myalgia	2 (13.3)	
		Weight loss	2 (13.3)	
		Increased serum bilirubin	1 (6.7)	
		Fatigue	1 (6.7)	
		Increased ALT	1 (6.7)	
		Hepatomegaly	1 (6.7)	
	High + High (4)	Anorexia	3 (75)	
		Increased ALP	3 (75)	
		Increased ALT	2 (50)	
		Increased serum bilirubin	1 (25)	
		Abdominal pain	1 (25)	
		Ascites	1 (25)	
		Hepatomegaly	1 (25)	
Pyrazinamide – Rifampin (19)	Low + Low (15)	Anorexia	4 (26.7)	Monitoring of LFTs at baseline and at 2, 4, 6, and 8 weeks of treatment. Patient education about reporting symptoms of liver injury
		Abdominal pain	4 (26.7)	
		Fever	3 (20)	
		Pale	3 (20)	
		Nausea	3 (20)	
		Weakness	3 (20)	
		Increased ALP	3 (20)	
		Myalgia	2 (13.3)	
		Weight loss	2 (13.3)	
		Increased serum bilirubin	1 (6.7)	
		Fatigue	1 (6.7)	
		Increased ALT	1 (6.7)	
		Hepatomegaly	1 (6.7)	
	High + High (4)	Anorexia	3 (75)	
		Increased ALP	3 (75)	
		Increased ALT	2 (50)	
		Increased serum bilirubin	1 (25)	
		Abdominal pain	1 (25)	
		Ascites	1 (25)	
		Hepatomegaly	1 (25)	

ACE inhibitors, angiotensin converting enzyme inhibitors; ALP, alkaline phosphatase; ALT, alanine aminotransferase; BUN, blood urea nitrogen; FBS, fasting blood sugar; HbA1c, glycated hemoglobin (A1c); LFTs, liver function tests; RBS, random blood sugar.

a Percentage was calculated based on dose categories.

## Discussion

DDIs remain one of the important therapeutic challenges in hospitalized patients (Zwart-van-Rijkom et al., 2009). The overall prevalence of pDDIs in our study was higher (62.3%) as compared to the prevalence among patients with other diseases such as HIV (52.2%)(Santos et al., 2016), liver cirrhosis (21.5%) (Franz et al., 2012), and hypertension (48%) (Subramanian et al., 2018). Moreover, our prevalence was lower in comparison with studies in patients with hemodialysis (89.1%) (Al-ramahi et al., 2016), hematopoietic stem cell transplantation (82.5%) (Trevisan et al., 2015), and CKD (95.9%) (Olumuyiwa et al., 2017). Furthermore, the prevalence of major pDDIs in the present study was higher (40%) as compared to a study in patients with liver cirrhosis (21.4%) (Franz et al., 2012). A similar prevalence has been reported in patients with hepatitis C (30–44%) (Kondili et al., 2017), whereas a higher prevalence has been reported in patients with stroke (61%) (Caratozzolo et al., 2016). The prevalence of contraindicated pDDIs in the present study was lower (6.6%) as compared to the prevalence reported by another study among patients with hepatitis C (16.7%) (Patel et al., 2015). These inconsistencies may be attributed to variability in the study design, study population, drug-prescribing patterns, considering types of pDDIs, and drug interaction screening software. Regardless of all these variations, our findings indicated a higher prevalence of pDDIs. Taking into consideration the findings of this study, patients with UTIs are at higher risk for DDIs. In addition, the Pakistani population is more exposed to pDDIs because of irrational use of medicines and non-availability of clinical pharmacy services as well as DDI-screening systems in hospitals (Ismail et al., 2017). Therefore, DDIs may result in a variety of negative clinical consequences such as decreased clinical effectiveness, adverse effects, hospitalization, and prolongation of hospital stay (Ray et al., 2004; Juurlink et al., 2013; Khan et al., 2017). In order to reduce/manage DDIs in hospitals, some evidence-based strategies have been suggested such as screening of medication profiles for pDDIs (Moura et al., 2012), clinical pharmacist involvement in evaluating patient medication profiles for pDDIs (Vonbach et al., 2007; Hahn et al., 2013), procedure for structured assessment of pDDIs (van-Roon et al., 2005), and appraisal of pertinent laboratory investigations (Geerts et al., 2009; Zwart-van-Rijkom et al., 2009).

Healthcare professionals could focus on DDIs with severe adverse outcomes based on their severity and documentation levels. Our findings about a high prevalence of pDDIs with moderate severity and fair documentation are consistent with the findings of other studies among hospitalized patients (Ismail et al., 2013; Murtaza et al., 2016). Therefore, it is essential for healthcare providers to properly identify the type of pDDIs, as it is vital for clinical management of pDDIs, designing prophylactic measures for their prevention, and reducing their associated risks.

Polypharmacy has become a serious concern among hospitalized patients with UTIs. These patients receive multiple therapies for treating comorbidities or associated complications (Dhodi et al., 2014; Panayappan et al., 2017). In literature, a positive relationship has been reported between pDDIs and polypharmacy, which is also indicated by our findings (Van-Leeuwen et al., 2013; Murtaza et al., 2016; Ismail et al., 2017). In our study, a significant association of pDDIs with various comorbidities such as DM, IHD, and CCF are mainly due to the prescription of drugs having high potential for DDI (Straubhaar et al., 2006; Mateti et al., 2011; Amin and Suksomboon, 2014). Salicylates, i.e., aspirin displaces sulfonylureas from protein binding sites, increasing the pharmacological effect and hypoglycemic risk of sulfonylureas (Kubacka et al., 1996). Drugs such as loop diuretics, non-steroidal anti-inflammatory drugs, angiotensin converting enzyme inhibitors (ACEIs), cyclosporine, or aminoglycosides deteriorate the renal status of the patient or compete for renal excretion of metformin, thus altering metformin concentrations in the body, which may change pharmacological response or cause adverse events (Graham et al., 2011). Similarly, some of the most common drug classes involved in DDIs in patients with cardiac diseases are anti-platelets, anticoagulants, anti-hypertensive, ACEIs, and diuretics, while the most common interacting drug pairs in patients with cardiac diseases are heparin-aspirin, aspirin-furosemide, aspirin-clopidogrel, aspirin-captopril, clopidogrel-heparin, clopidogrel-torsemide, and heparin-warfarin (Mateti et al., 2011). Healthcare professionals should have knowledge about possible risk factors for pDDIs, so that patients at risk should be carefully individualized in order to optimize therapy and prevent or minimize the incidence of DDIs.

The clinical relevance of DDIs emphasizes the significance of medications profile screening for DDIs, which is also supported by published studies (Vonbach et al., 2007; Geerts et al., 2009; Ismail et al., 2017). Potential adverse effects of DDIs have been related to clinical manifestations of the patients in the present study. Such methods are helpful for healthcare professionals to monitor the associated adverse outcomes of interactions. Our findings indicate that adverse events were more prevalent among patients taking higher doses of prescribed drugs. Adverse events associated with interactions can be minimized by monitoring patients’ signs/symptoms and lab tests. Therefore, proper consideration should be awarded to this aspect of therapy. Another considerable strength of our study is the provision of a list of most frequent DDIs, including their management strategies and monitoring parameters. Not all identified pDDIs are clinically important. Therefore, there is an immense need to develop a list of clinically important interactions that are observed in hospitalized patients with UTIs. This list will be used by healthcare professionals for selective identification and management of pDDIs in order to develop clinical guidelines and to prevent adverse consequences related to DDIs. As guardians of patient safety and health, healthcare professionals are responsible for identifying and preventing adverse consequences associated with DDIs (Harrington et al., 2011). A clinician’s knowledge about DDIs can decrease the occurrence of adverse drug effects, provide good quality care, and prevent associated medico-legal issues. Most of these interactions are preventable and can be managed by considering alternative therapy and dose reduction.

Following are the potential limitations of this study. This study was conducted at tertiary care hospitals, where patients with UTIs are mainly admitted for the management of complications associated with UTIs and comorbid disease. The pDDIs that we have identified are mainly related to the use of medicines for the management of such problems. Our results may not be generalizable to outpatient settings where the disease and the drug interaction pattern may be different. Furthermore, we have used the term pDDIs, as we could not confirm the causal linkage. We only correlated the potential adverse consequences of interactions with patients’ clinical features. While data concerning negative clinical consequences caused by DDIs are scarce, some retrospective studies are available in the published literature, highlighting the importance of clinical relevance of interactions (Ray et al., 2004; Juurlink et al., 2013).

## Conclusions

PDDIs are highly prevalent among patients with UTIs. Software-based screening of pDDIs is recommended in order to identify, prevent/reduce, and manage pDDIs in UTI patients. Knowledge about the most frequent pDDIs could help healthcare professionals to prevent DDIs and their associated adverse outcomes. In patients with UTIs, polypharmacy, DM, IHD, and CCF increase the risk of interactions. The prevalence of adverse events is greater among patients taking higher doses of interacting drugs. Careful monitoring for adverse events associated with pDDIs will contribute to patient safety. Most of the interactions can be managed by considering alternative therapy and dose reduction.

## Data Availability

The datasets used and/or analyzed during the current study are available from the corresponding author on request.

## Ethics Statement

Ethical approval was obtained from Institutional Research and Ethics Board of the Postgraduate Medical Institute, Peshawar. As this was a retrospective study based on the clinical data maintained in the record room, therefore informed consent was not applicable, according to our institutional guidelines.

## Author Contributions

SN and MI contributed to the design of the study. SN and FK collected data, organized the data, and contributed to data analysis. SN and MI wrote the first draft of this manuscript. SN, MI, and FK contributed to manuscript drafting and revision. All authors approved the submitted version of the manuscript.

## Conflict of Interest Statement

The authors declare that the research was conducted in the absence of any commercial or financial relationships that could be construed as a potential conflict of interest.

## Abbreviations

ACEIs, angiotensin converting enzymes inhibitors; ALP, alkaline phosphatase; ALT, alanine aminotransferase; AMAs, antimicrobial agents; ATC, anatomical therapeutic chemical classification; ATD, alternate day; BD, twice a day; BP, blood pressure; BUN, blood urea nitrogen; CCF, congestive cardiac failure; CI, confidence interval; CKD, chronic kidney disease; DDIs, drug-drug interactions; DM, diabetes mellitus; FBS, fasting blood sugar; HbA1c, glycated hemoglobin (A1c); IHD, ischemic heart disease; IQR, interquartile range; LFTs, liver function tests; OD, once a day; OR, odds ratio; pDDIs, potential DDIs; RBS, random blood sugar; UTIs, urinary tract infections.
