# Supplementary material for: Potential Drug-Drug Interactions in Patients With Urinary Tract Infections: A Contributing Factor in Patient and Medication Safety
Source: Front Pharmacol. 2019 Sep 17;10:1032. doi: 10.3389/fphar.2019.01032 (PMC6758591; doi:10.3389/fphar.2019.01032)
Supplement: Supplementary file 1 [file Table_1.docx]

**SUPPLEMENTARY TABLE 1** Description of the widespread clinically important potential drug–drug interactions in patients with UTIs

| **Drug Interaction** | **Frequency** | **Severity** | **Documentation** | **Adverse outcomes** |
| --- | --- | --- | --- | --- |
| Aspirin – Insulin | 42 | Moderate | Fair | Hypoglycemia |
| Insulin – Metformin | 33 | Moderate | Fair | Hypoglycemia |
| Insulin – Ramipril | 24 | Moderate | Fair | Hypoglycemia |
| Aspirin – Nitroglycerin | 21 | Moderate | Good | Increase in nitroglycerin concentrations and additive platelet function depression |
| Aspirin – Ramipril | 20 | Moderate | Fair | Decreased ramipril effectiveness |
| Aspirin – Bisoprolol | 20 | Moderate | Good | Increased blood pressure |
| Aspirin – Furosemide | 20 | Major | Good | Reduced diuretic effectiveness and possible nephrotoxicity |
| Calcium containing products – Ceftriaxone | 19 | Contraindicated | Good | Formation of ceftriaxone-calcium precipitates and is contraindicated in neonates. |
| Isoniazid – Rifampin | 19 | Major | Good | Hepatotoxicity |
| Pyrazinamide – Rifampin | 19 | Major | Good | Hepatotoxicity |
| Insulin – Valsartan | 18 | Moderate | Fair | Hypoglycemia |
| Aspirin – Clopidogrel | 16 | Major | Fair | Increased risk of bleeding |
| Aspirin – Spironolactone | 15 | Major | Good | Reduced diuretic effectiveness, hyperkalemia, or possible nephrotoxicity |
| Insulin – Sitagliptin | 14 | Moderate | Fair | Hypoglycemia |
| Bisoprolol – Insulin | 14 | Moderate | Good | Hypoglycemia or hyperglycemia; decreased symptoms of hypoglycemia |
| Furosemide – Ramipril | 13 | Moderate | Good | Postural hypotension (first dose) |
| Insulin – Losartan | 12 | Moderate | Fair | Hypoglycemia |
| Aspirin – Dexamethasone | 12 | Moderate | Good | Increased risk of gastrointestinal ulceration and subtherapeutic aspirin serum concentrations |
| Clopidogrel – Omeprazole | 11 | Major | Excellent | Reduced plasma concentrations of clopidogrel active metabolite and reduced antiplatelet activity |
| Dexamethasone – Rifampin | 11 | Moderate | Good | Decreased dexamethasone effectiveness |
