# Supplementary material for: Potential Drug-Drug Interactions in Patients With Urinary Tract Infections: A Contributing Factor in Patient and Medication Safety
Source: Front Pharmacol. 2019 Sep 17;10:1032. doi: 10.3389/fphar.2019.01032 (PMC6758591; doi:10.3389/fphar.2019.01032)
Supplement: Supplementary file 2 [file Table_2.docx]

**SUPPLEMENTARY TABLE 2** Most frequently prescribed antimicrobial agents among patients with UTIs

| **Class of drugs (ATC code)^a^** | **Drugs** | **Frequency** |
| --- | --- | --- |
| Second, third, and fourth generation cephalosporins (J01DC^b^, J01DD^c^, J01DE^d^) | Cefoperazone | 260 |
|  | Ceftriaxone | 137 |
|  | Cefpodoxime | 27 |
|  | Cefepime | 24 |
|  | Cefixime | 11 |
|  | Cefotaxime | 10 |
|  | Cefuroxime | 1 |
|  | Ceftazidime | 1 |
| Beta-lactam antibacterial, penicillin (J01C) | Sulbactam | 258 |
|  | Amoxicillin | 21 |
|  | Piperacillin | 14 |
|  | Ampicillin | 11 |
|  | Cloxacillin | 10 |
|  | Tazobactam | 1 |
|  | Benzyl penicillin | 1 |
| Antimalarial (P01B) | Artesunate | 119 |
|  | Quinine | 12 |
|  | Lumefantrine | 9 |
|  | Artemether | 9 |
|  | Chloroquine | 2 |
|  | Primaquine | 1 |
|  | Hydroxychloroquine | 1 |
| Drugs for treatment of tuberculosis (J04A) | Rifampin | 19 |
|  | Pyrazinamide | 19 |
|  | Isoniazid | 19 |
|  | Ethambutol | 18 |
|  | Streptomycin | 4 |
| Intestinal anti-infective (A07A) | Metronidazole | 44 |
|  | Rifaximin | 16 |
|  | Vancomycin | 3 |
|  | Miconazole | 3 |
|  | Moxifloxacin | 6 |
|  | Levofloxacin | 2 |
|  | Ofloxacin | 1 |
|  | Norfloxacin | 1 |

-ATC, anatomical therapeutic chemical classification

-^a^ Drugs were grouped in accordance with the Anatomical Therapeutic Chemical Classification System.

-^b^ J01DC ATC code is for second generation cephalosporin.

-^c^ J01DD ATC code is for third generation cephalosporin.

-^d^ J01DE ATC code is for fourth generation cephalosporin.
