# Supplementary material for: Potential Drug-Drug Interactions in Patients With Urinary Tract Infections: A Contributing Factor in Patient and Medication Safety
Source: Front Pharmacol. 2019 Sep 17;10:1032. doi: 10.3389/fphar.2019.01032 (PMC6758591; doi:10.3389/fphar.2019.01032)
Supplement: Supplementary file 3 [file Table_3.docx]

**SUPPLEMENTARY TABLE 3** Most frequently prescribed drugs (besides AMAs) among patients with UTIs

| **Class of drugs (ATC code)^a^** | **Drugs** | **Frequency** |
| --- | --- | --- |
| Drugs used in diabetes (A10) | Insulin | 245 |
|  | Metformin | 47 |
|  | Gliclazide | 26 |
|  | Sitagliptin | 18 |
|  | Glimepiride | 12 |
|  | Vildagliptin | 7 |
|  | Glyburide | 4 |
|  | Pioglitazone | 3 |
| Analgesic (N02) | Paracetamol | 211 |
|  | Tramadol | 46 |
|  | Aspirin | 1 |
| Proton pump inhibitor (A02BC) | Omeprazole | 149 |
|  | Esomeprazole | 85 |
|  | Rabeprazole | 5 |
| Electrolyte solution (B05XA) | Sodium chloride | 215 |
|  | Potassium chloride | 15 |
|  | Sodium bicarbonate | 3 |
| Vitamins (A11) | Multivitamin | 90 |
|  | Vitamin D | 46 |
|  | Pyridoxine | 18 |
|  | Vitamin B | 9 |
|  | Vitamin E | 1 |
| Diuretics (C03) | Furosemide | 64 |
|  | Spironolactone | 34 |
|  | Hydrochlorothiazide | 19 |
| Glucocorticoids (H02AB^b^, R03BA^c^) | Dexamethasone | 69 |
|  | Beclometasone | 22 |
|  | Prednisolone | 16 |
|  | Hydrocortisone | 8 |
| Drugs for constipation (A06A) | Lactulose | 37 |
|  | Sodium picosulfate | 29 |
|  | Sodium biphosphate/sodium phosphate | 25 |
|  | Lactitol | 6 |
|  | Mannitol | 1 |
| H2-receptor antagonists (A02BA) | Ranitidine | 63 |
|  | Loratadine | 1 |

-ATC, anatomical therapeutic chemical classification

-^a^ Drugs were grouped in accordance with the Anatomical Therapeutic Chemical Classification System.

-^b^H02AB ATC code is for dexamethasone, prednisolone, and hydrocortisone.

-^c^ R03BA ATC code is for beclometasone.

- Dimenhydrinate (antiemetic) was among the most frequently prescribed drugs, but not found in ATC system.
